# Supplementary material for: Use of Amplified Lewy Body Dementia Fibrils and Autoradiography to Characterize Binding of Radioligand Tg-1-90B to Alpha-Synuclein Fibrils in Postmortem Brain Tissue
Source: Cells. 2025 Sep 22;14(18):1477. doi: 10.3390/cells14181477 (PMC12468313; doi:10.3390/cells14181477)

Use of amplified Lewy body dementia fibrils and autoradiography to characterize binding of radioligand Tg-1-90B to alpha-synuclein fibrils in postmortem brain tissue

Jennifer Y. O'Shea[1†], Dhruva D. Dhavale[1†], Helen Hwang[1], Zachary Smith[1], Thomas J.A. Graham[2], Robert H. Mach[2], Paul T. Kotzbauer[1]*

[1] Department of Neurology and Hope Center for Neurological Disorders, Washington University School of Medicine, St. Louis, MO, 63110, USA

[2] Department of Radiology, Perelman School of Medicine, University of Pennsylvania, Philadelphia, PA 19104, USA

† These authors contributed equally to this work.

* Correspondence: kotzbauerp@wustl.edu

Supplementary Figure 1: [^{3}H]Tg-1-90B saturation binding assays with synthetic Aβ and recombinant Tau fibrils. Saturation binding assays were performed as described and total along with non-specific binding (NSB) were analyzed by global fit non-linear regression analysis in GraphPad. (A) The average binding affinity (K_d) of [^{3}H]Tg-190B to Aβ fibrils was 31 ± 1.7 nM with an average Bmax of 8.6 ± 2.4 pmol/nmol. (B) The average binding affinity (K_d) of [^{3}H]Tg-190B to recombinant Tau fibrils was 48.7 ± 13 nM with an average Bmax of 7.5 ± 0.6 pmol/nmol. Representative curves are shown and data points represent mean +/- s.d. Similar results were obtained from n = 2 independent assays.

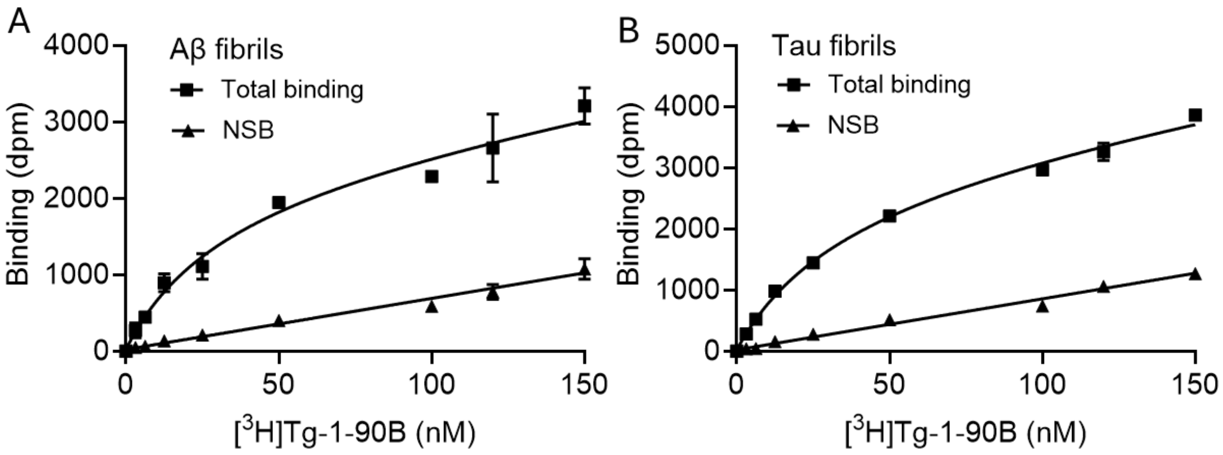

Supplementary Figure 2: [^{3}H]Tg-1-90B autoradiography with AD tissue and anti-Aβ immunostaining. (A-B) Silver halide crystals in the nuclear emulsion are activated by [^{3}H]Tg-1-90B, resulting in dense silver grain accumulation that co-localize with Aβ plaques identified by immunofluorescence with anti-Aβ antibody, mHJ3.4 (red). (C-D) Addition of 500 nM of excess unlabeled Tg-1-90B displaced radioligand [^{3}H]Tg-1-90B, resulting in minimal silver grain accumulation over Aβ plaques as identified by immunofluorescence with anti-Aβ antibody, mHJ3.4 (red). Scale bar = 20 nm and n = 2 slides.

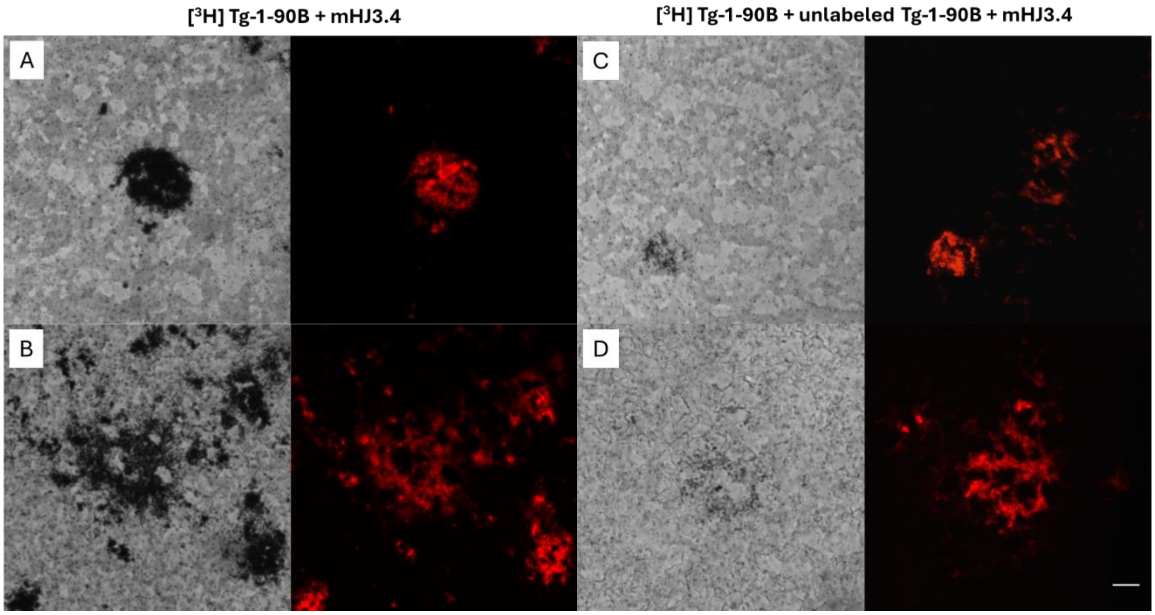

Supplementary Figure 3: [^{3}H]Tg-1-90B autoradiography with AD tissue and anti-tau immunostaining. (A-B) Lack of silver halide crystal accumulation on neurofibrillary tangles (which contain tau fibrils), identified by immunofluorescence with anti-tau antibody, PHF1(green). Representative classic neurofibrillary tangles are shown by red arrows. Scale bar = 10 nm and n = 2 slides.

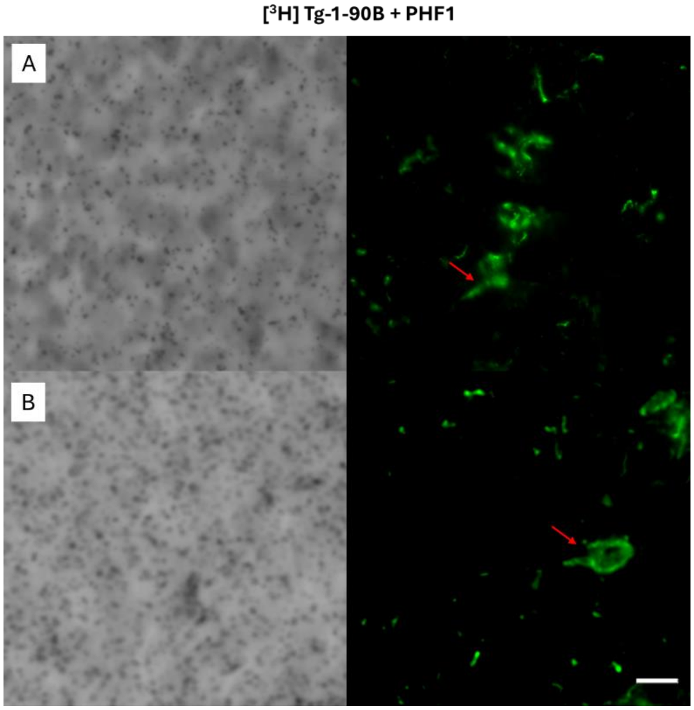

Supplementary Figure 4: [^{3}H]Tg-1-90B autoradiography with control tissue and anti-Asyn immunostaining. (A) No specific or consistent pattern of silver halide crystal accumulation was observed on control tissue sections incubated with [^{3}H]Tg-1-90B. Control tissue was immunostained with anti-Asyn antibody P-syn/81A, which confirmed the absence of Asyn pathology. Scale bar = 20 nm and n = 2 slides.

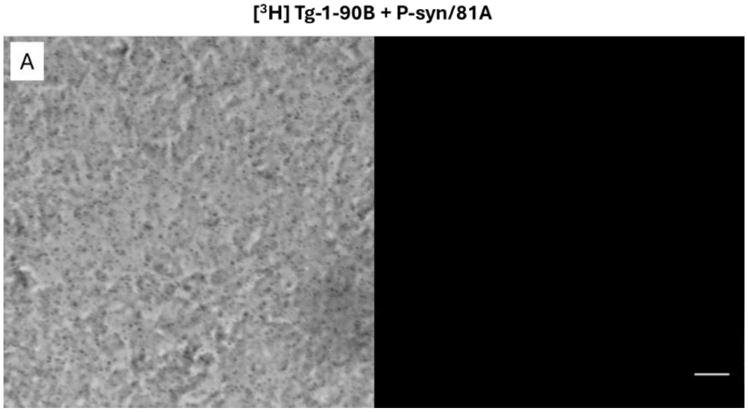

Supplement: Supplementary file 1 [file cells-14-01477-s001.zip › cells-3842293-supplementary.pdf]
